# Automated Stabilization, Enhancement and Capillaries Segmentation in Videocapillaroscopy

**DOI:** 10.3390/s23187674

**Published:** 2023-09-05

**Authors:** Vincenzo Taormina, Giuseppe Raso, Vito Gentile, Leonardo Abbene, Antonino Buttacavoli, Gaetano Bonsignore, Cesare Valenti, Pietro Messina, Giuseppe Alessandro Scardina, Donato Cascio

**Affiliations:** 1 Department of Mathematics and Informatics, University of Palermo, 90128 Palermo, Italy; vincenzo.taormina@unipa.it (V.T.); cesare.valenti@unipa.it (C.V.); 2 Department of Physics and Chemistry, University of Palermo, 90128 Palermo, Italy; giuseppe.raso@unipa.it (G.R.); vitogentile@live.it (V.G.); leonardo.abbene@unipa.it (L.A.); antonino.buttacavoli@unipa.it (A.B.); gaetano.bonsignore@unipa.it (G.B.); 3 Department of Surgical Oncological and Stomatological Disciplines, University of Palermo, 90127 Palermo, Italy; pietro.messina01@unipa.it (P.M.); alessandro.scardina@unipa.it (G.A.S.)

**Keywords:** capillaroscopy, microcirculation, video stabilization, signal enhancement, capillaries segmentation, U-net, deep learning

## Abstract

Oral capillaroscopy is a critical and non-invasive technique used to evaluate microcirculation. Its ability to observe small vessels in vivo has generated significant interest in the field. Capillaroscopy serves as an essential tool for diagnosing and prognosing various pathologies, with anatomic–pathological lesions playing a crucial role in their progression. Despite its importance, the utilization of videocapillaroscopy in the oral cavity encounters limitations due to the acquisition setup, encompassing spatial and temporal resolutions of the video camera, objective magnification, and physical probe dimensions. Moreover, the operator’s influence during the acquisition process, particularly how the probe is maneuvered, further affects its effectiveness. This study aims to address these challenges and improve data reliability by developing a computerized support system for microcirculation analysis. The designed system performs stabilization, enhancement and automatic segmentation of capillaries in oral mucosal video sequences. The stabilization phase was performed by means of a method based on the coupling of seed points in a classification process. The enhancement process implemented was based on the temporal analysis of the capillaroscopic frames. Finally, an automatic segmentation phase of the capillaries was implemented with the additional objective of quantitatively assessing the signal improvement achieved through the developed techniques. Specifically, transfer learning of the renowned U-net deep network was implemented for this purpose. The proposed method underwent testing on a database with ground truth obtained from expert manual segmentation. The obtained results demonstrate an achieved Jaccard index of 90.1% and an accuracy of 96.2%, highlighting the effectiveness of the developed techniques in oral capillaroscopy. In conclusion, these promising outcomes encourage the utilization of this method to assist in the diagnosis and monitoring of conditions that impact microcirculation, such as rheumatologic or cardiovascular disorders.

## 1. Introduction

Capillaroscopy is a particular non-invasive microscopic diagnostic technique that allows the analysis of the body’s capillaries (study of the microcirculation) using a capillaroscope [1]. The technique provides video-making images of the capillaries, which are fundamental for the study of microcirculation [2]. The analysis of the microcirculation is of particular interest as alterations in the capillaroscopic picture can represent the only evidence of the presence of an early-stage disease. Morphological and densitometric changes can affect systemic and non-systemic diseases such as rheumatoid arthritis, lichen planus, diabetes, pemphigus and pemphigoid, hypercholesterolemia, Sjögren’s syndrome and scleroderma [1,2]. Capillaroscopy is employed in various areas of the body, including the fingertips, skin, tongue, and oral cavity. In particular, capillaroscopy of the oral cavity has been recognized as a particularly useful method for investigating capillary microcirculation in the nailfold bed, given the oral mucosa’s easy accessibility and repeatability [3,4,5].

During oral capillaroscopy examinations, healthcare professionals can observe and assess the structure and morphology of capillaries present on the lips, buccal mucosa, vestibular masticatory/gingival mucosa, and other specific sites. This technique enables the detection of microvascular alterations that may be indicative of various pathological conditions such as diabetes, hypertension, peripheral arterial occlusive disease, systemic sclerosis, and other conditions that can affect microcirculation [3,6].

In capillaroscopic imaging, the quality of acquired images plays a crucial role in the accuracy and reliability of subsequent analysis [6]. However, capillaroscopic images often suffer from various sources of noise, artifacts, and distortions. These issues can impact the effectiveness of image analysis and hinder the interpretation of results. Therefore, preprocessing of capillaroscopic images is essential to enhance their quality and facilitate accurate analysis.

Image preprocessing techniques for capillaroscopy involve a series of steps aimed at improving image quality and removing unwanted artifacts. These steps may include noise reduction, contrast enhancement, image normalization, and removal of motion blur or other distortions [7]. By applying these preprocessing techniques, the visibility of capillary structures can be improved, enabling more accurate segmentation and analysis of capillaroscopic images. Another important aspect of capillaroscopic imaging is the need for frame stabilization [8]. Capillaroscopic examinations often require magnification and high-resolution imaging, which can lead to increased sensitivity to motion and micro-movements of the subject [9]. Even slight movements can cause significant blurring and misalignment of capillary structures, making accurate analysis challenging.

To address this issue, researchers have been actively exploring methods for frame stabilization in capillaroscopic imaging [8]. These methods aim to minimize the impact of subject movements and ensure that capillary structures remain in focus and aligned throughout the imaging process. Various approaches, including physical stabilization using brackets or cuffs, as well as digital video stabilization algorithms, have been investigated to mitigate the effects of motion and improve the quality of capillaroscopic images [10]. The development of effective frame stabilization techniques is crucial in capillaroscopy, as it can significantly enhance the accuracy and reliability of subsequent analysis [11]. By minimizing motion-induced artifacts and blurring, stabilized frames enable more precise segmentation and quantitative measurements of capillary parameters [12].

These phases of improvement and stabilization of the capillaroscopic images are also essential to make an automatic segmentation process of the microcirculation effective. Segmentation of capillary loops is a critical step in capillaroscopy image analysis, as it enables the extraction of quantitative measures of capillary morphology and function. These measures include capillary density, diameter, length, tortuosity, and blood flow velocity, which can be used for the diagnosis and monitoring of microvascular diseases. Various segmentation algorithms have been proposed for capillaroscopic images, including thresholding-based methods, region-growing-based methods, and machine learning-based methods such as convolutional neural networks [13].

Once the capillary loops are segmented, they can be analyzed to extract quantitative measures of capillary morphology and function. These measures can be used to assess the effectiveness of treatments and interventions and to evaluate the progression of the disease. For example, capillary density and blood flow velocity have been shown to be useful in monitoring the effects of vasodilator therapy in patients with systemic sclerosis [14]. Similarly, capillary density and tortuosity have been shown to be predictive of microvascular complications in patients with type 1 diabetes [15].

The following paragraph outlines related works and the challenges that motivated us to develop an automated system to assist in the analysis of oral cavity microcirculation.

## 2. Related Works

Capillaroscopy is a non-invasive imaging technique that allows visualization of the microcirculation in vivo, and it has been widely used in the diagnosis and monitoring of microcirculation-related diseases such as Raynaud’s phenomenon, scleroderma, and systemic lupus erythematosus [16,17]. However, the quality of the images captured using capillaroscopes is often affected by noise, artifacts, and low contrast, which can compromise the accuracy of the subsequent analysis. Automated stabilization and processing of capillaroscopy images could greatly enhance the accuracy and efficiency of microcirculation analysis and aid in the early detection and treatment of microcirculation-related diseases. To address this challenge, several works have proposed preprocessing techniques to improve the quality of capillaroscopic images.

In [8], the authors propose a new automated method for detecting and counting white blood cells (WBCs) in nailfold capillary images. The method uses deep learning to segment the capillaries, video stabilization based on FFT (Fast Fourier Transform) to reduce motion artifacts, and WBC event detection algorithms to identify the WBCs.

The study of Doshi et al. [18], given the significant influence of image denoising and enhancement during the preprocessing stage on subsequent analysis, focuses on evaluating the performance of five enhancement techniques specifically designed for capillary skeletonization. The techniques under investigation include the α-trimmed filter, bilateral filter, bilateral enhancer, anisotropic diffusion filter, and non-local means. The authors report the visual and quantitative performance on a series of different capillaroscopic images.

Oharazawa et al. [19] develop an algorithm capable of effectively handling regions by employing color component separation techniques as preprocessing, followed by blood vessel extraction filtering. The method uses independent component analysis (ICA) and the Frangi filter to extract capillary regions. To validate the accuracy of capillary blood vessel extraction, the resulting images were compared with ground truth data.

In [20], the authors presented a novel approach to compare images obtained from a microscope. The objective is to explore various feature extraction techniques such as template matching and SIFT (Scale Invariant Feature Transform). The focus of the investigation lies in the comparison between previous and real-time capillary images. By employing template matching, the authors perform a real-time comparison between the captured image of the target capillary and the previously acquired image, regardless of the magnification.

Nirmala et al. [21] proposed an image processing approach to measure the dimensions of capillaries. To achieve this, the authors employ image processing algorithms to identify the optimal enhancement filters and segmentation methods for accurately segmenting the capillaries. Specifically, they used Wiener and bilateral filters to improve image quality. Furthermore, they applied morphological operations to detect and segment capillary boundaries.

In the field of capillaroscopy, the research activity in the field of nail capillaroscopy has been relatively richer. In this context, previous research has primarily focused on reducing small-scale movements by physically stabilizing the finger and the resulting video footage [22,23]. One common approach to achieve physical stabilization is the utilization of a metal bracket, which effectively minimizes finger movements in relation to the microscope. However, despite the use of the bracket, there may still be subtle finger movements, as noted by Watanabe et al. [22]. Digital video stabilization has emerged as an effective technique for reducing motion artifacts in capillaroscopy videos. However, the quality and computational efficiency of different stabilization algorithms can vary significantly [24]. Various digital video stabilization methods have been employed in capillaroscopy, including optical flow, phase correlation, cross-correlation, feature-based, intensity-based, and block-matching algorithms utilizing metrics such as mutual information, cross-correlation coefficient and mean squared error (MSE) [24,25,26,27]. However, limited studies have thoroughly validated the outcomes of stabilization algorithms, specifically in the context of capillaroscopy. Wu et al. [28] and Dobbe et al. [25] indirectly validated their stabilization methods by assessing subsequent capillaroscopy measurements.

In recent years, there has been growing interest in developing automated segmentation methods for microvasculature structures observed from medical images. Hwang et al. [29] propose an automated method for blood vessel detection and segmentation, using the Mask R-CNN framework, for thrombus analysis. The authors designed the utilization of a combined approach involving complete intersection over union (CIoU) and smooth L1 loss to ensure accurate blood vessel detection. Segmentation is further improved with a modified focal loss. The authors evaluated their method on 60 patient studies, achieving the highest F1 score (92%).

Several studies have proposed automated segmentation methods for microcirculation structures from capillaroscopy images. In [30], the authors proposed for nailfold capillary images the utilization of a deep neural network with a Res-Unet architecture. To train the network, they utilized a dataset comprising 30 nailfold capillary images. Subsequently, the network was evaluated on a separate dataset containing 20 images, resulting in the generation of a binarized map. The obtained results showcased a mean accuracy of 91.72%.

Liu et al. [31] introduced a novel deep learning architecture called DAFM-Net, designed to achieve accurate segmentation. To enhance the convergence ability of the deep neural network, the authors incorporated group normalization as an effective normalization technique. To validate the effectiveness of the proposed model, they conducted ablation studies and segmentation experiments. The results demonstrate that when compared to the ground truth, the method achieves a Dice score of 87.34%.

Mahmoud et al. [32] introduced a novel two-step image processing algorithm aimed at functionally analyzing microscopic images in an automated manner. Their approach involves leveraging a trained Convolutional Neural Network (CNN) to perform this task. The first step of our algorithm employs a modified vessel segmentation algorithm to extract the precise location of vessel-like structures within the images. Subsequently, in the second step, a 3D-CNN is utilized to assess whether these identified vessel-like structures exhibit blood flow or not. The accuracy obtained was equal to 83%.

Much less investigated is capillaroscopy of the oral cavity. In this context, Tutuncu et al. [33] presented an analysis of segmentation methods known in the literature as Otsu, Region Growing, Fuzzy C-means, Fast Marching and H-minimum. The accuracy results obtained for the various methods implemented ranged between 44% and 97%. The best segmentation accuracy was obtained with the H-minimum method.

In their work, Bellavia et al. [6] employed wavelet analysis and mathematical morphology as preprocessing techniques on the images. They then applied segmentation to minimize lighting variations between capillary and background images. The result in terms of the Jaccard index was equal to 85.8%.

Spera et al. [34] presented a methodology for identifying and extracting regions of interest corresponding to capillaries in the oral mucosa. The extracted features are crucial for accurate diagnosis purposes in real-time applications. To achieve this, a discrete version of the wavelet transform is utilized to segment the images obtained from video sequences captured using a prototype capillaroscope. Subsequently, a comprehensive set of appropriate characteristics is automatically computed to enable a precise evaluation of the peripheral microcirculation. However, the greatest limitation of this work is that a quantitative analysis of the results was not carried out, but only a qualitative one.

In this work, we addressed the challenges related to the movement of the acquisition probe in capillaroscopy and the difficulty in tracking/analyzing the microcirculation in the oral cavity by presenting an automated system for processing videocapillaroscopy based on a stabilization algorithm and a signal enhancement phase. Additionally, an automatic capillary segmentation phase was implemented with a dual objective: quantitatively evaluate the signal improvement achieved through the developed techniques and automatically segment the microcirculation in the oral cavity. For this purpose, the well-known deep convolutional network U-net was employed using transfer learning. The performance of the automated system was quantified by comparing the automatic segmentations with the ground truths from a public database.

## 3. Materials and Methods

The relationship between the details of blood microcirculation (such as capillary geometry and velocities of red blood cells—RBCs) and certain potential diseases requires capturing a sequence of clear images depicting the flow of RBCs in capillaries. This process involves creating a dataset of images that enables a comparison between patients diagnosed with known pathologies using other techniques and healthy individuals.

**Figure 1:**
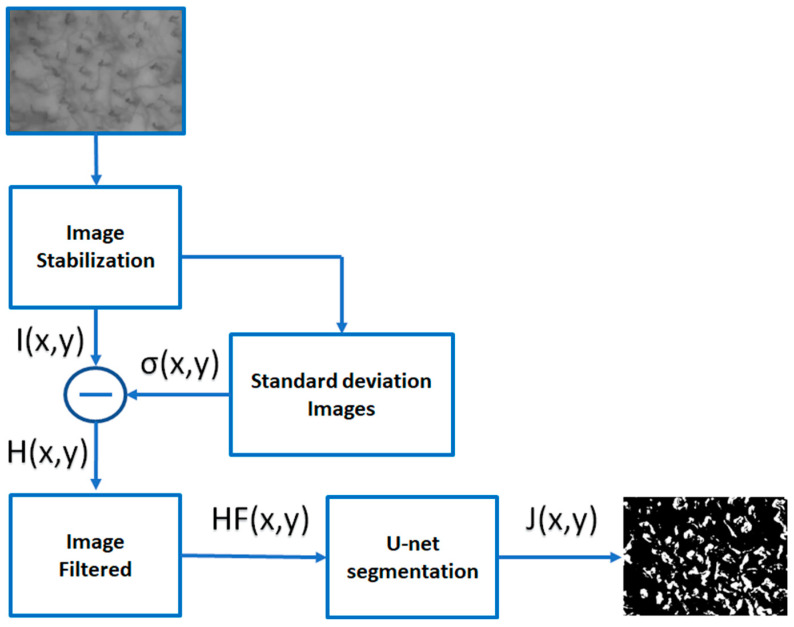
Flow chart of the proposed method. After the image stabilization step, the average image is obtained using a temporal neighborhood of frames containing the image itself. The difference between the stabilized image and the standard deviation image is made in order to emphasize the motion of the blood cells over time. The images thus obtained are then filtered to obtain images that highlight the regions where the most accentuated RBC movements are located. Finally, the last phase performs a segmentation process that isolates the capillaries involved in RBC motion.

In this section, we will describe the experimental setup used for video sequences of the capillary blood flow registration and signal enhancement. The noise and the very low contrast between the background and capillaries of the images obtained from video capillaroscopy of the oral cavity requires a processing phase accomplished by transforming and analyzing the image in order that capillaries appear enhanced. Figure 1 shows the flow chart of the proposed method. In the following subsections, the phases that make up the method will be discussed in detail.

As will be discussed in Section 3.2, each image of a capillaroscopy video is subject to motion artifacts occurring due to the movement of the patient and/or the probe. In nailfold capillaroscopy, this problem is minimal since the use of a metal bracket almost eliminates finger movement. Conversely, in oral capillaroscopy, the movement of the probe inside the oral cavity causes the capillary to move considerably within the resulting video frame, reducing the accuracy of measurements, especially during a magnification of 150× or greater. In this case, to reduce motions in the capillaroscopy video, it is necessary to use stabilization algorithms. The outcome of the application of these stabilization algorithms is a sequence of stabilized frames, which allows a clearer analysis of the motion of the erythrocytes. The signal enhancement step, described in detail in Section 3.3, analyzes the static part and the dynamic part of the signal in order to separate the microvasculature from the background. As far as capillaroscopy is concerned, the background is the portion of tissue not crossed by RBCs, which represents the static part of the image.

### 3.1. Optical System Setup and Database

The video-capillaroscope (VCS) Horus (Adamo srl, Trapani, Italy) used in this study consists of:

- a central unit with a cold halogen 100 W light source whose luminosity and white balancing can be adjusted automatically or manually by a control device;
- an optical/digital probe connected to the central unit by a fiber bundle 2 m long;
- a high-resolution color micro-television camera equipped with a high magnification (up to 500×) zoom lens system;
- a high-resolution personal computer with a dedicated graphics card connected to the central unit through a S-video cable;
- a real-time analog to DV converter connecting the central unit to a secondary personal computer used to record images at 120 frames per second with a spatial resolution of 640 × 480 pixels at an 8-bit grayscale.

Since manual positioning of the probe requires contact with the inside of the mouth, very high magnifications were avoided as they caused excessive instability of the videos produced and, therefore, the impossibility of following the blood flow. For this reason, the videos used in this work were acquired by the operator with magnifications of 150×.

The development of automatic diagnostic support systems in medical imaging is closely linked to the collection of a database of selected images [35,36,37]. For the development of the method proposed here, a database obtained from a cohort of thirty-two patients was used. They participated in the research study by giving their consent for capillaroscopic examination and for the use of their personal medical information for scientific purposes. The study strictly followed the privacy regulations and guidelines outlined by Italian laws concerning the handling of personal data. The medical professionals in our research team conducted a comprehensive examination of the mucosal regions encompassing the upper and lower lips, as well as the left and right buccal mucosae. Additionally, they assessed the vestibular masticatory/gingival mucosa of the II and V sextants.

### 3.2. Stabilization

**Figure 2:**
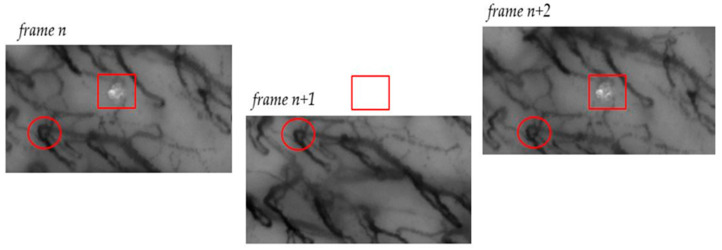
The figure presents a notable example illustrating the effect of motion during frame acquisition. On the left, in frame *n*, an artifact is indicated by a square, while a section of a blood vessel is marked with a circle. In the center, the *n* + 1 frame shows a visible downward shift in relation to the reference points (square and circle). The same portion of the blood vessel marked by the circle remains visible, but the artifact is now out of frame and no longer observable. In the right frame, frame *n* + 2, both markers are once again within the frame, indicating an upward motion of the probe.

A capillaroscopic video captures frames of a specific area within the oral cavity over a defined time interval. The portion recorded at time *t*_0_, represented by the *n*-th frame, and the portion captured at time *t*_1_, represented by the (*n* + 1)-th frame, may exhibit misalignments, and these misalignments can be quite significant. Multiple factors contribute to the motion observed between consecutive frames, including movements of the acquisition probe (such as translation and rotation) and the patient’s movements (for instance, caused by breathing). Figure 2 illustrates an example of such misalignment, visually emphasizing the magnitude of this issue.

**Figure 3:**
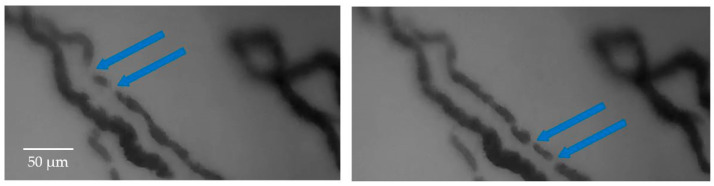
The movement of RBCs is evident when comparing two frames captured at different time points. The intermittent flow of RBCs results in a partial visualization of the general capillary, leading to variations in the acquisition of vessels between successive frames. The arrows in the Figure indicate examples of this partial visualization of the capillary in consecutive frames.

An additional challenge in the frame stabilization process arises from the dynamic displacement of RBCs along the capillaries. Typically, RBCs flow continuously within the blood vessels, resulting in a continuous representation of the vessel itself (indirectly obtained from the presence of RBCs). Therefore, the movement of RBCs is not visible from frame to frame, considering the sampling frequency used by the oscilloscope. However, in finer capillaries, the motion of RBCs can become discontinuous, leading to variations between successive frames and resulting in a stroke-like visualization of the capillaries. Figure 3 illustrates the movement of RBCs in two consecutive captured frames, highlighting how a capillary appears differently in these frames.

In computer vision, the video stabilization process employs a variety of techniques to minimize the impact of camera movement on the resulting footage. It relies on estimating the motion of captured objects within each frame. In this study, effective stabilization ensures the alignment of consecutive frames, specifically targeting the primary structures, namely the body’s capillaries. Single unchained RCBs present in the microvasculature, representing moving objects within the scene, should be disregarded during the stabilization process. However, after the stabilization phase, these single unchained erythrocytes play a crucial role in reconstructing the finest capillaries.

To enhance the effectiveness and robustness of the stabilization phase, the input video was divided into segments whenever the probe’s speed (and consequently the relative motion between frames) exceeded a threshold value. This decision was influenced by the acquisition method employed. During the acquisition phase, the operator focuses on specific areas of the oral cavity, attempting to keep the probe still on the region of interest for a few seconds before moving it to another area. The significant and sudden shift between regions, and thus the frames acquired within that time interval, are not subject to analysis.

**Figure 4:**
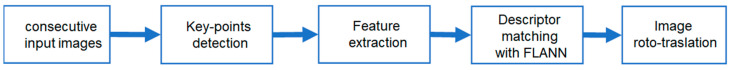
Flow chart of the stabilization method features-based implemented.

In the literature, various families of digital stabilization methods are available, including block-matching, optical flow, and feature-based methods [38]. In this study, the feature-based approach was chosen due to its computational efficiency compared to other methods, given the satisfactory frame rate achievable in videocapillaroscopy of the oral cavity (120 frames/s). This approach has the potential to enable near real-time medical applications despite the high frame rate. While this type of solution is not new to medical imaging, its application to the specific problem addressed is novel. This method operates by detecting and tracking features such as corners or edges in a sequence of frames. By tracking these features over time, the camera motion can be estimated. The feature-based approach, whose flowchart is shown in Figure 4, involves several steps, including keypoints detection, feature extraction, descriptor matching and motion estimation.

**Figure 5:**
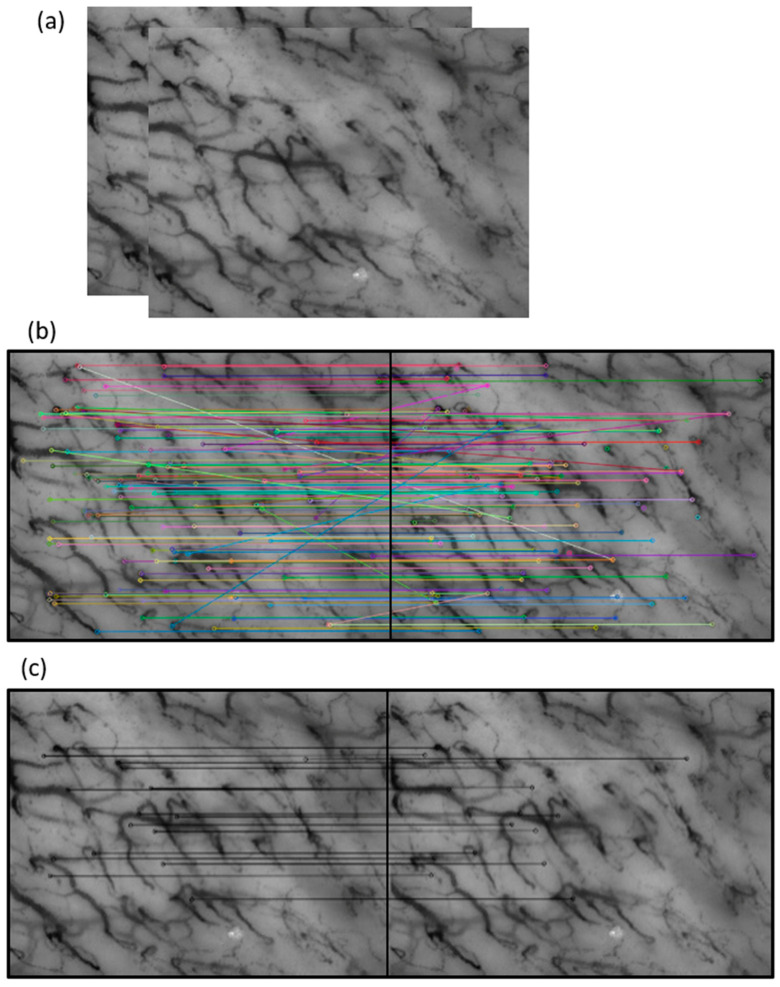
Key steps to find the keypoints of the method: (**a**) two consecutive frames; (**b**) in each frame, the key points are identified, and their characteristics are extracted, then all the correspondences between the keypoints found by the procedure are identified; (**c**) the accurately matching keypoints are selected by applying a threshold to the match score.

The SURF (SpeedUp Robust Features) algorithm has been implemented as a detector of keypoints and as a feature descriptor [39]. Figure 5 highlights the key steps to find keypoints. The ability to identify keypoints between two frames enables motion estimation. However, it is important to note that there may be incorrect matches or keypoints detected on moving objects, such as unchained RCBs. To mitigate this issue, a substantial number of keypoints need to be extracted. For this purpose, we have developed an iterative procedure in order to extract at least ten keypoints from each frame; the process continues until the number of keypoints detected in each analyzed frame exceeds this lower limit. In our specific problem, in the matching phase, most of the matches are related to fixed structures such as capillaries, and only a few matches will concern moving objects in the scene (not concatenated globules) or artifacts. To limit the matches of non-fixed structures, a threshold on the match score has been applied. The matching method implemented in this work is based on FLANN (Fast Library for Approximate Nearest Neighbor Search) [40].

For easy repeatability of the method, we report below the pseudocode of the developed stabilization algorithm (Algorithm 1); it was implemented in C++ with the VS2010 IDE, making use of the OpenCV [41] library.
**Algorithm 1.** Pseudocode of Stabilization AlgorithmTake *frame-n* and *frame-n*+1 set *hessian_threshold* to 1000 #detect keypoints in *frame_n* and in *frame_n*+1 while (*hessian_threshold* > 0) ‘and’ (keypoints in *frame_n* ‘or’ in *frame_n*+1 are < 10)   detect the keypoints using SURF on the two frames   set *hessian_threshold* to *hessian_threshold* − 20 #extract descriptors calculate SURF descriptors (feature vectors) from keypoints in *frame_n* and in *frame_n*+1 #matching the *frame_n* and *frame_n*+1 keypoints matching descriptor vectors using FLANN matcher #check matches number If size of matches < 5    Exit and print “matches are too few” #Search good match calculate min distances between keypoints take the good match if the distance is less than *max*(2*min_dist, 0.02) # 0.02 is a small arbitrary value in the event that min_dist is very small #check good match number, if too few calculate with a less stringent value If good match keypoints < 5    take the good match if distance is less than *max*(3*min_dist, 0.03) #check good match number, if too few take the best four match If good match keypoint < 5    take the best 4 match as good match keypoints #discard good match that are clearly out of average calculate movement for every good match calculate mean and standard deviation of movements for every good match   if good match movement is out of twice the deviation standard       delete good match #motion estimation with good match calculate translation and rotation motion between *frame_n* and *frame_n*+1

**Figure 6:**
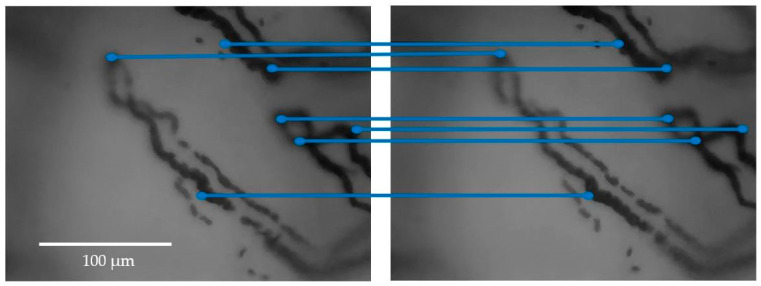
An example of the application of the stabilization method to two consecutive frames of videocapillaroscopy, with the keypoints highlighted. For better clarity, only parts of the frames containing few capillaries are presented, rather than the entire frames. The blue lines graphically show the couplings between keypoints.

Figure 6 shows an example of application of the stabilization method implemented to two consecutive frames in which the couplings between keypoints are highlighted.

After locating the corresponding keypoints in consecutive frames, a perspective transformation can be applied, and motion estimation is evaluated. With motion estimation, the stitching technique can be used to generate a larger image by merging the pixel values of overlapping parts while maintaining the pixel values without overlap.

**Figure 7:**
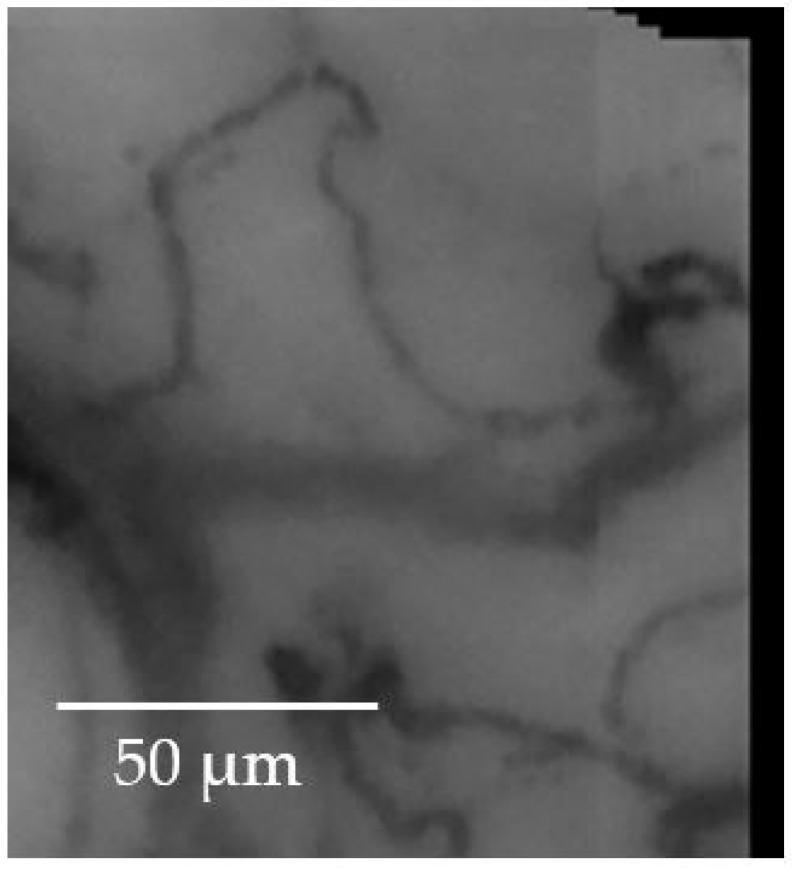
The stitching of some frames with a cropped corner to highlight both the overlapping and non-overlapping portions of the image.

Figure 7 highlights the stitching of some frames; to make the overlapping and the non-overlapping portions perceived, the crop in a corner has been taken.

### 3.3. Signal Enhancement Process

Once the stabilization phase of the frames has been carried out, it is possible to improve the content of the signal (the microcirculation), for example, by reconstructing parts of missing capillaries or emphasizing capillaries that are not very visible, in order to correctly segment the microcirculation. The phase described in this subsection, therefore, has the objective of improving the signal-to-noise ratio, and this passes through the identification in the most suitable way of the continuous structure of the capillaries after stabilization. To this end, the video frames were averaged over time. The arithmetic mean *B*(*i*,*j*,*k*) for a set of n consecutive frames of intensity value *I*(*i*,*j*,*k*) for each discrete pixel coordinate (*i*,*j*) is given by:

(1) $$B\left(i,j,k\right)=\frac{1}{2n+1}\sum_{k^{\prime }=k-n}^{k+n}I(i,j,k^{\prime })$$

where *k* is the current video frame number of a full video frames set containing N frames (N total number of video frames), while 2*n* + 1 represents the time environment under analysis, in terms of number of frames. The video acquisition rate being 120 frames per second and considering the characteristic recovery time of the regions to be around one second, we chose a value of n equal to 5. This allowed us to analyze an interval of 11 frames for each image. Obviously, if *k* is smaller than n or greater than N − *n*, then the average must be calculated only on the frames with *k*′ ∈ [0, N].

As suggested by Japee et al. [42], a video of the microcirculation contains a notable quantity of information. To obtain the best visibility of the capillary network with regard to its geometry and perfusion by RBCs, the authors proposed different two-dimensional visualization techniques. In our study, we used the standard deviation images properly calculated as a useful tool capable of enhancing the video signal. The areas where the variance is small correspond typically to tissue regions, whereas the regions with high variance contain capillaries having numerous RBCs flowing through there. The flow of operations implemented in this work for signal enhancement is the one already presented in Figure 1. The standard deviation is then found as follows:

(2) $$\sigma \left(i,j,k\right)=\sqrt{\frac{1}{2n+1}\sum_{k^{\prime }=k-n}^{k+n}{\left[I\left(i,j,k^{\prime }\right)-B(i,j,k)\right]}^{2}}$$

The standard deviation image is subsequently subtracted from the specific frame.

(3) $$H\left(i,j,k\right)=I\left(i,j,k\right)-\sigma \left(i,j,k\right)$$

**Figure 8:**
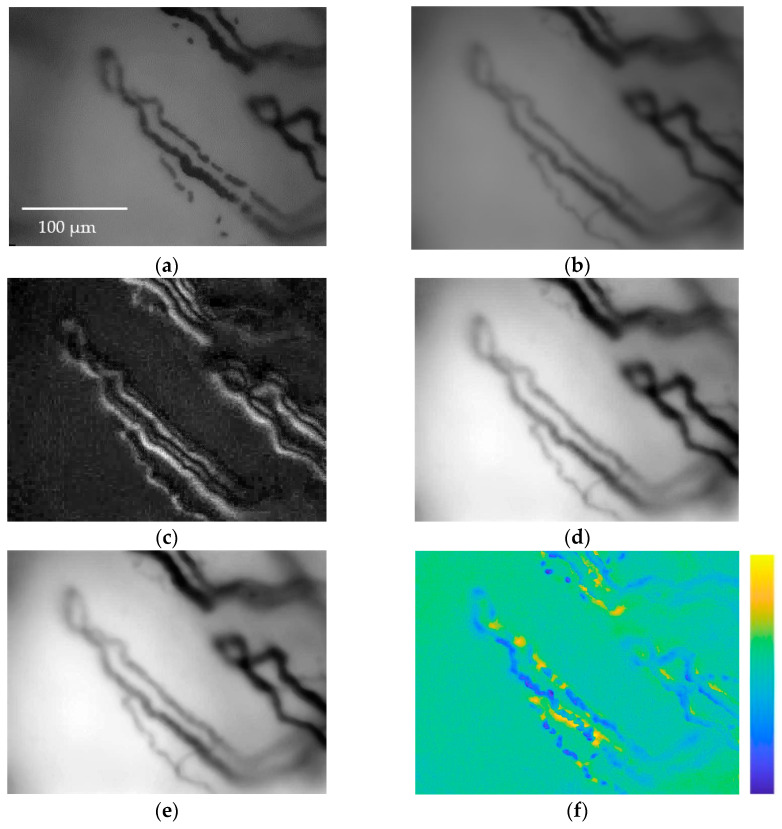
(**a**) Frame *I*(*i*,*j*,*k*) showing RBCs flowing along capillaries of a subject; (**b**) arithmetic mean *B*(*i*,*j*,*k*); (**c**) standard deviation *σ*(*i*,*j*,*k*); (**d**) *H*(*j*,*j*,*k*) subtraction between *I*(*i*,*j*,*k*) and *σ*(*i*,*j*,*k*), the image is normalized; (**e**) image *HF*(*i*,*j*,*k*) after applying the median filter, the image is normalized. It is easy to see how parts of the capillaries missing inframe *I*(*i*,*j*,*k*) are clearly reconstructed in *HF*(*i*,*j*,*k*). (**f**) The difference between the original frame *I*(*i*,*j*,*k*) and the enhanced frame *HF*(*i*,*j*,*k*) is presented in false color to easily appreciate the results achieved via the image enhancement method.

**Figure 9:**
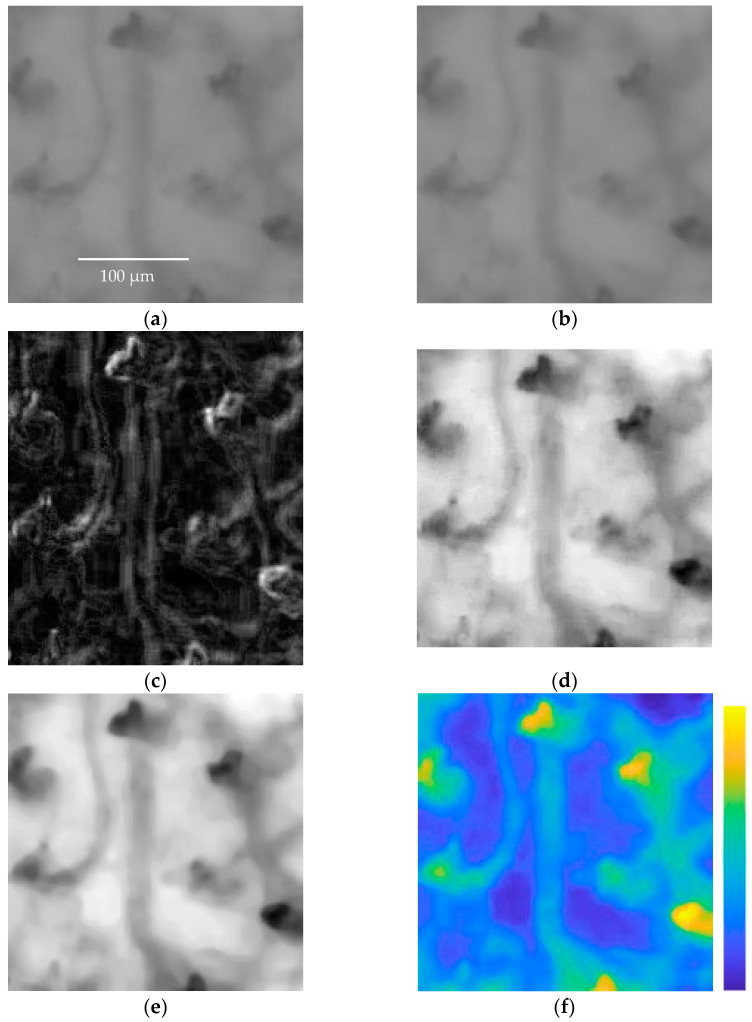
(**a**) Frame *I*(*i*,*j*,*k*) showing RBCs flowing along capillaries of a subject; (**b**) arithmetic mean *B*(*i*,*j*,*k*); (**c**) standard deviation *σ*(*i*,*j*,*k*); (**d**) *H*(*j*,*j*,*k*) subtraction between *I*(*i*,*j*,*k*) and *σ*(*i*,*j*,*k*), the image is normalized; (**e**) image *HF*(*i*,*j*,*k*) after applying the median filter, the image is normalized. It is easy to see how the blood vessels in frame *I*(*i*,*j*,*k*) are more clearly visible in the *HF*(*i*,*j*,*k*) image. (**f**) The difference between the original frame *I*(*i*,*j*,*k*) and the enhanced frame *HF*(*i*,*j*,*k*) is presented in false color to easily appreciate the results achieved via the image enhancement method.

The resulting *H* matrix values are then shifted to ensure positive values, followed by normalization to ensure intensity values fall within the range of [0, 255]. Finally, a median filter with a 3 × 3 convolution mask was applied to reduce noise. Figure 8 and Figure 9 show two examples of applications of the enhancement method presented.

As can be seen from Figure 8 and Figure 9, the procedure allows for a much better appreciation of the vessels, reconstructing missing parts of capillaries or emphasizing vessels that are not very visible. Clearly, the capillaroscopic frames after the enhancement process can lead to a more effective segmentation of the microvasculature.

### 3.4. Capillaries Segmentation U-Net Based

**Figure 10:**
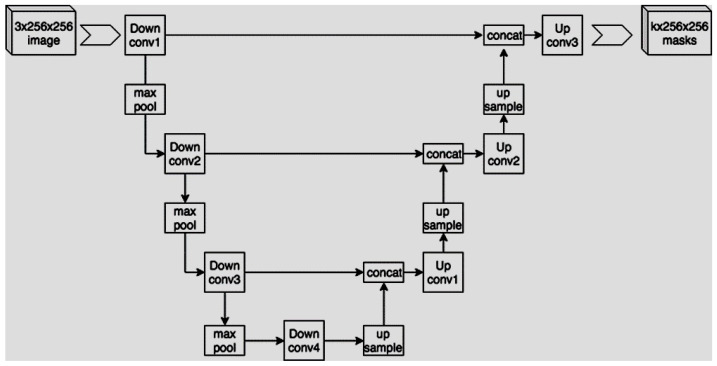
U-shaped scheme.

The previous phases of the proposed methodology aimed to enhance the signal to accurately identify the microcirculation in the oral cavity. Subsequently, a capillary segmentation procedure based on deep learning was implemented, also with the objective of quantitatively evaluating the preceding stages. We, therefore, want to evaluate the degree of similarity between manual segmentation and automatic segmentation for the cases for which we have the ground truth. The goal of our work was to implement an algorithm to segment input images in order to obtain binary masks, where white pixels represent areas with capillaries. To this end, we decided to implement a U-Net-based approach. U-Net is a convolutional neural network developed by Ronneberger et al. in 2015 [43], and it was specifically developed for biomedical image segmentation. More specifically, this network’s structure is made up of a contracting path consisting of a typical convolutional network with a series of repeated applications of convolutions, each followed by a rectified linear unit (ReLU) and a max pooling operation. Then, a second expansive path consists of a sequence of up-convolutions and concatenations with high-resolution features from the contracting path. These two paths are usually represented with the characteristic U-shaped scheme shown in Figure 10.

During the contraction phase, the spatial information is reduced while the feature information is enhanced. Conversely, the expansive pathway combines both the feature and spatial information, increasing the output resolution. This results in a final output that has the same dimensions as the input image. We implemented a U-Net architecture using Keras, consisting of four contracting blocks with 32, 64, 128, and 256 convolutional filters, respectively. Similarly, we employed four expanding blocks with the same filter sizes. The models were designed to accept grayscale images with a size of 160 × 160 pixels as input, where each pixel’s gray value ranged from 0 to 1. The output produced was also of size 160 × 160, with values converted to binary form.

#### 3.4.1. Dataset and Data Preparation

In order to test the implemented methods, the database of intra-oral capillaroscopic images presented in the research titled “A non-parametric segmentation methodology for oral videocapillaroscopic images” (Bellavia et al. [6]) was used. The authors had obtained ethical approval from the Ethics Committee of the Policlinico Hospital of Palermo (Italy) [6,34]. Proper correspondence was established with the authors to obtain the necessary images. It was necessary to use this database for the test phase of the developed method as this database is accompanied by a ground truth, i.e., the optimal segmentation masks, in this case, produced as a synthesis of the segmentation of different physicians. Indeed, the authors obtained 620 × 476 sized images labeled by 6 different domain experts, each of whom superimposed white pixels on those areas where capillaries were present. The 6 binary masks thus obtained were then summarized, and a new average binary mask was constructed and used to realize the ground truth needed to evaluate automated algorithms. Overall, the database consists of 22 images captured with a capillaroscopy and related to 22 different patients. It is evident that the number of images available for evaluating the segmentation is certainly limited in any case. To make up for this deficiency, we applied a series of rotations to the input images by α angles, where α was sampled equally spaced in (0°, 360°). Since the U-Net requires input images of 160 × 160, we had to divide each image into smaller subregions, each of which becomes an input. From these images, we removed all of those with less than 200 white pixels. With all these augmentations and subdivisions, we obtained a total of 4334 images, representing the entire dataset we used for training.

For the various techniques implemented, the result obtained in terms of segmentation goodness was evaluated, obviously referring to the ground truth and quantitatively evaluating it by means of the Jaccard index. The Jaccard similarity coefficient [44], also known as Intersection over Union (IoU), is a numerical value in the range [0, 1], where 1 indicates a perfect segmentation between ground truth and automatic segmentation. This index is among the most used in quantifying the quality of segmentation, as it considers both the background and the foreground.

#### 3.4.2. Training

The U-Net described above was trained using the Keras API for 20 epochs, with the ADAM optimizer and weighted categorical cross-entropy as the loss function. We incorporated weights due to the imbalanced nature of the dataset, where black pixels outnumbered white pixels significantly. Hence, the weights were computed to be proportional to the number of white and black pixels. For evaluation, a Leave-One-Out Cross-Validation (LOOCV) procedure was implemented, involving 22 separate trainings. Each training utilized all the images, except one, as the training set, while the remaining image was used as the validation set. This approach was adopted to achieve more generalizable results, especially considering the limited number of original images available. Moreover, we also compared the results considering different preprocessing configurations:

- no preprocessing;
- enhancement;
- stabilization and enhancement.

**Figure 11:**
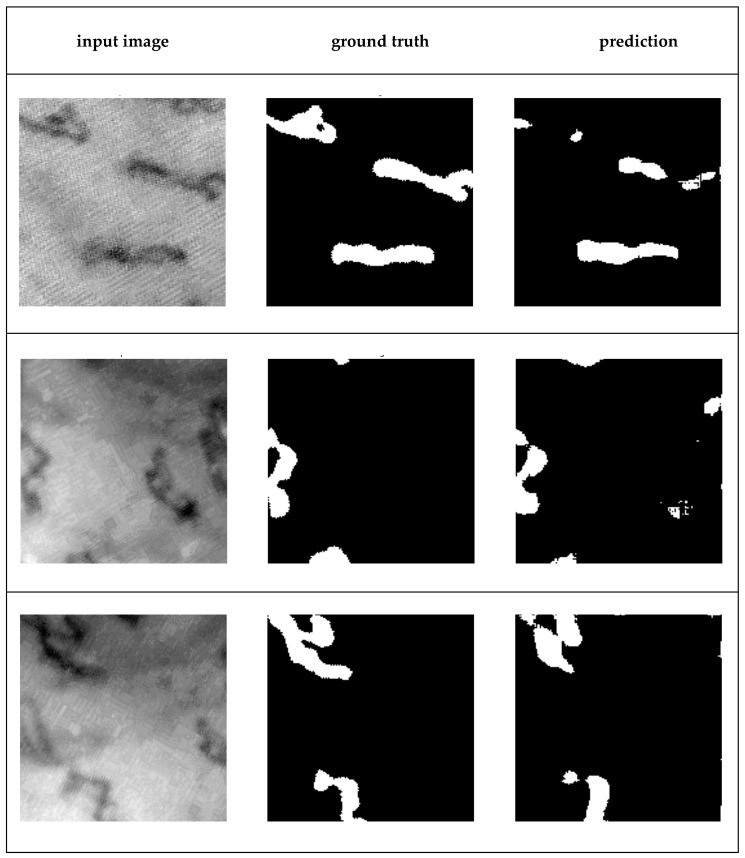
Three examples of capillaroscopy sub-images containing capillaries and their resulting segmentation. From left to right: input image, ground truth, prediction (automatic segmentation produced by the U-net trained).

In addition to the preprocessing configurations using the techniques proposed here, configurations with well-known techniques, particularly those already employed in capillaroscopy, have also been analyzed. These are presented in the following Section 3.5. Figure 11 shows three examples of segmentation of capillaries obtained with the U-net network trained with the method described here.

### 3.5. Alternative Comparative Techniques

In this section, we present the state-of-the-art techniques that we have implemented to enable a more effective evaluation alongside the techniques proposed here. The performance results of these additional techniques will be reported in the “Results” section.

#### 3.5.1. Stabilization

For a quantitative comparison of the proposed stabilization method, we employed a well-known approach based on Fast Fourier Transform (FFT), as introduced in [45]. This method calculates the motion between two frames by determining the translation vector that maximizes the cross-correlation between them. To achieve this, each frame is transformed into the frequency domain using FFT. Subsequently, the two images are element-wise multiplied, and the result is inverse-transformed into the spatial domain. This technique has also been used in the field of nailfold capillaroscopy [8]. In that context, the authors utilized labels obtained from the segmentation process rather than raw frames. For the implementation, we utilized the code provided by the authors in MATLAB [46]. This code iteratively registers two frames using selective oversampling through a Fourier transform multiplied with a matrix.

#### 3.5.2. Enhancement

For enhancement, we chose to compare the Contrast-Limited Adaptive Histogram Equalization (CLAHE) method [47]. This enhancement procedure has already been effectively employed in the field of capillaroscopy, as seen in [48]. In contrast to histogram equalization, CLAHE operates on small regions within the image, enhancing the contrast of each region by equalizing its histogram. Subsequently, the enhanced neighboring regions are merged using bilinear interpolation to eliminate artificially induced boundaries. The main parameter to be adjusted is the number of regions in which to divide the image. The implementation was carried out using MATLAB. The parameter value that yielded the best result was 64 regions.

#### 3.5.3. Segmentation

We conducted our analysis to include a comparative evaluation using the Mask R-CNN model with a ResNet-50-FPN backbone [49]. Mask R-CNN [50], used, for instance in segmentation problems and already used for the segmentation of blood vessels [29], is an extension of Faster R-CNN. In comparison to the latter, it introduces an additional third branch, for instance mask prediction. This operation runs in parallel with the two existing branches within Faster R-CNN (bounding box regressor and classifier). For each detected instance, Mask R-CNN provides the output class, its bounding box, and an overlaid binary mask. For the implementation, Detectron2 [51] was used, a framework created by Facebook AI Research, and implemented in Pytroch. Specifically, the R-CNN mask has been configured with the “COCO-InstanceSegmentation/mask_rcnn_R_50_FPN_3x.yaml” configuration, as provided by Detectron2′s model zoo [52]. We fine-tuned the solver configuration to process batches of 2 images with a learning rate of 0.00025. Additionally, during the training, we specified the regions of interest (ROI) heads to handle a batch size of 512 per image, tailored for a single-class segmentation problem. Both the tested methods (U-Net and Detectron2) displayed competent segmentations. However, in a consistent manner, our original U-Net approach manifested superior performance in terms of accuracy and Jaccard’s index. This deepened comparative analysis further accentuates the efficacy of our proposed U-Net model, shedding light on its relative strengths over prevalent techniques. The method used for training data processing is the same used for U-net and described in Section 3.4.2.

## 4. Results

In this section, we report the results in terms of the goodness of segmentation obtained for the capillaries. Table 1 shows the segmentation results obtained for the different configurations explored.

**Figure 12:**
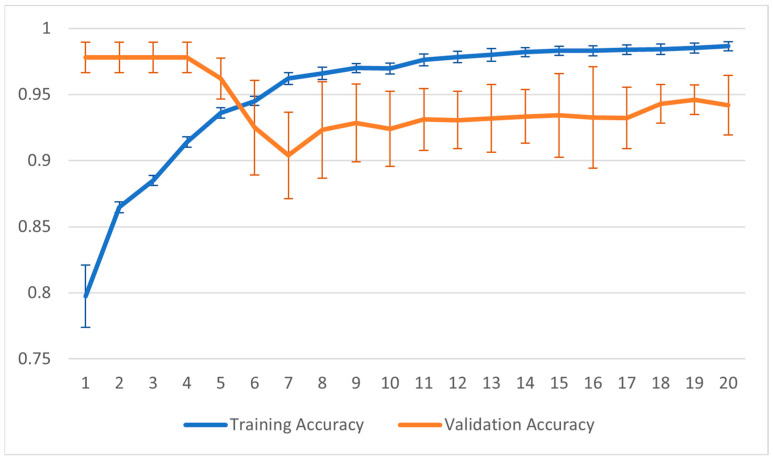
Graph of accuracy during the training process.

**Figure 13:**
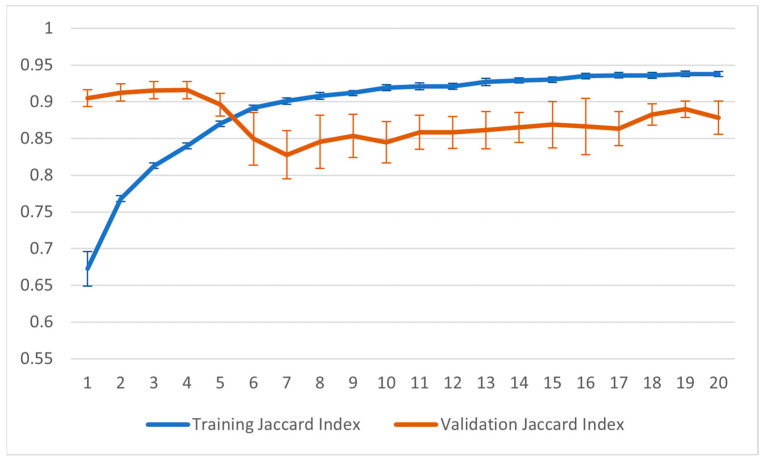
Graph of Jaccard index during the training process.

Figure 12 and Figure 13 show the training graphs for the Jaccard index and the accuracy for the configuration that reported the best result in Table 1. The values reported in the graphs represent the averages obtained from the 22 sessions of training carried out using the LOOCV method. For this training, the point of minimum validation loss during the training was obtained in seven epochs.

To obtain a more objective assessment of the effectiveness of the implemented techniques and, consequently, the achieved outcomes, Table 2 presents a performance comparison with the two existing methods in the literature for oral cavity microcirculation segmentation. It is important to highlight that the performance values in the table are derived from the analysis of the same database across all studies. For a more direct comparison with the aforementioned works, we have also computed the following performance metrics: sensitivity, specificity, and accuracy.

Finally, the configuration that yielded the best result in Table 1 was evaluated using the mask R-CNN segmentation method, obtaining a Jaccard index of 90.0% and an accuracy of 96.3%.

## 5. Discussion

The outcomes presented in Table 1 unmistakably illustrate the efficacy of the stabilization process in achieving noteworthy enhancement. The implemented stabilization and enhancement techniques have notably contributed to a statistically significant improvement in the segmentation process. Notably, the techniques proposed in this study, both for enhancement and stabilization, demonstrated performance that is certainly not inferior to the respective state-of-the-art methods (CLAHE and FFT-based stabilization). Statistically, it cannot be asserted that they are superior due to an error magnitude of approximately 4% observed in both accuracy and the Jaccard index. Although with a low statistical significance, we can say that the proposed temporal analysis technique, in the specific problem of blood vessel reconstruction, allows a better signal-to-noise ratio than the CLAHE technique.

The results obtained with the techniques presented in this work in comparison with other methods presented in the literature and presented in Table 2 are certainly encouraging. In particular, the result obtained in terms of the Jaccard index is significantly better than that obtained in [6]. Despite employing data augmentation and training with the LOOCV method, the performance results are still influenced by the limited number of test data available in the public database. This limitation hinders a comprehensive appreciation of the performance results concerning the effectiveness of the implemented techniques, both for preprocessing and segmentation. This is probably the reason why our best result is not significantly better than the one obtained by Tutuncu et al. [33]; however, in terms of sensitivity (ability to locate capillaries), our method reported a better result. We add to the discussion that it is important to acknowledge that accuracy is not an ideal figure of merit for quantifying the segmentation of biomedical images, particularly when the foreground is significantly smaller than the background, as observed in this case. Finally, it is worth noting that the two segmentation methods used (U-net and mask R-CNN) yielded nearly identical results. Certainly, expanding the test database could allow for better statistics (reduced error associated with the results) as well as greater model robustness.

## 6. Conclusions

In this paper, we have presented techniques for stabilizing, enhancing and segmenting capillaroscopic images.

These techniques are of paramount importance in ensuring accurate assessments of the microvasculature, offering invaluable insights into a diverse array of pathological conditions. Despite capillaroscopy’s established status as an imaging modality, the quality of the resultant images can frequently be compromised by a multitude of factors. Challenges such as the inherently low contrast within the capillary network, unwelcome motion artifacts, and suboptimal lighting conditions often plague the fidelity of the images. As a result, several methods have been developed to address these limitations and improve the image quality for more accurate analysis. The techniques discussed in this paper include image stabilization, signal enhancement, noise reduction, data augmentation, and leave-one-out cross-validation for the training procedure. Additionally, we have presented a method for capillary segmentation based on a deep learning approach. The deep network implemented allowed the comparison between the result of automatic segmentation and that produced by experts in relation to the problem. A quantitative performance evaluation was carried out, both for the techniques presented and for the techniques of comparison of the state of the art. In particular, the stabilization with the keypoints matching method based on FLANN (Fast Library for Approximate Nearest Neighbor Search) and the enhancement process through the temporal analysis of the standard deviations allowed the best segmentation result, Jaccard index equal to 90.1%.

It is noteworthy that the primary objective of these endeavors is the enhancement of diagnostic and monitoring capabilities for a range of conditions impacting microcirculation, encompassing rheumatologic and cardiovascular disorders. Employing capillaroscopy within the oral cavity holds the potential to offer a promising avenue for early identification and understanding of oral diseases, potentially culminating in improved treatment results and patient well-being.

Additionally, encouraging from the results obtained, our future research activities on the subject could concern the targeted application of the system developed to support the diagnosis of specific diseases diagnosable by means of oral capillaroscopy, such as autoimmune diseases or rheumatological diseases.

## Figures and Tables

**Table 1:** Segmentation results.

Configuration	Jaccard Index
No preprocessing	84.1%
Enhancement (CLAHE based)	85.9%
Enhancement (our method)	86.8%
Stabilization + Enhancement (FFT + CLAHE based)	88.3%
Stabilization + Enhancement (our methods)	90.1%

**Table 2:** Performance comparison.

Method	Jaccard Index	Sensitivity	Specificity	Accuracy
Tutuncu et al. [33]	-	81.1%	98.4%	96.7%
Bellavia et al. [6]	85.8%	-	-	-
Our Method	90.1%	85.3%	96.9%	96.2%

## Data Availability

Not applicable.
